# Prognosis and Predictor Factors of Permanent Pacemaker Implantation after Transcatheter Aortic Valve Replacement: A Retrospective Analysis of the Post-Transcatheter Aortic Replacement Clairval Hospital Registry

**DOI:** 10.3390/jcm13113050

**Published:** 2024-05-22

**Authors:** Vassili Panagides, Emna Sakka, Edouard Cheneau, Ahmed Bouharaoua, Jacques Vicat, Elisabeth Leude-Vaillant, Philippe Rochas, Frédéric Collet, Marie-Paule Giacomoni

**Affiliations:** 1 Service de Cardiologie, Ramsay Santé, Hôpital Privé Clairval, 13009 Marseille, France; vassili.panagides@gmail.com (V.P.); emna.sakkacuvelier@ramsaysante.fr (E.S.); edouard.cheneau@gmail.com (E.C.); bouharaoua13@yahoo.fr (A.B.); philip.rochas@gmail.com (P.R.); fredcharles.collet@gmail.com (F.C.); 2 Service de Chirurgie Cardiaque, Ramsay Santé, Hôpital Privé Clairval, 13009 Marseille, France; jacques.vicat@orange.fr (J.V.); secretariatvaillant@gmail.com (E.L.-V.)

**Keywords:** TAVR, permanent pacemaker implantation, bundle branch block, conduction disturbances, valve replacement

## Abstract

**Background/Objectives**: Despite procedural improvements, post-transcatheter aortic valve replacement (TAVR) conduction disorders remain high. Analyzing the data from a monocentric TAVR registry, this study aims to determine predictive factors for PPI (primary outcome), the indication for PPI, and long-term outcomes among these patients (secondary outcomes). **Methods**: Conducted at Clairval Hospital in Marseille, France, this retrospective study included all consecutive patients from June 2012 to June 2019. Clinical, electrocardiographic, echocardiographic, and procedural data were collected, with outcomes assessed annually. Logistic regression identified PPI predictors and survival analyses were performed. **Results**: Of the 1458 patients initially considered, 1157 patients were included. PPI was needed in 21.5% of patients, primarily for third-degree atrioventricular block (46.4%). Predictor factors for PPI included baseline right bundle branch block (ORadj 2.49, 95% CI 1.44 to 4.30; *p* = 0.001), longer baseline QRS duration (ORadj 1.01, 95% CI 1.00 to1.02, *p* = 0.002), and self-expandable valves (ORadj 1.82, 95% CI, 1.09 to 3.03; *p* = 0.021). Seven-year estimated mortality was higher in PPI (43.3%) vs. non-PPI patients (30.9%) (log rank *p* = 0.048). PPI was an independent predictive factor of death (ORadj 2.49, 95% CI 1.4 to 4.3; *p* = 0.002). **Conclusions**: This study reveals elevated rates of PPI post-TAVR associated with increased mortality. These results underscore the pressing necessity to refine our practices, delineate precise indications, and enhance the long-term prognosis for implanted patients.

## 1. Introduction

Aortic stenosis (AS) is the most common valvular heart disease in developed countries. In recent years, transcatheter aortic valve replacement (TAVR) has emerged as a method of choice for treating AS in patients considered unfit for cardiac surgery. The accumulation of operators’ expertise and technological enhancement has significantly decreased the procedure complications. However, cardiac rhythm disorders remain a cause of concern and are now regarded as the most prevalent complication occurring in up to 20% of patients post-TAVR [1]. In France, the FRANCE 2 national registry reported permanent pacemaker implantation (PPI) rate of 15.6% for patients who operated between 2010 and 2012 [2], while the FRANCE TAVI registry reported a rate of PPI of 17.5% between 2013 and 2015 [3]. Several studies attempted to determine factors predisposing to PPI [4]. Unfortunately, the occurrence of this complication appears to be highly variable and influenced by electrical factors (pre-existing conduction abnormalities), anatomical factors, and procedural factors (balloon valvuloplasty and implantation depth). Despite recent expert consensus papers [5,6], the medical management of post-TAVR conduction disorders continues to be debated with significant variability between centers [7]. The present study aimed to evaluate, in a large monocentric cohort of consecutive patients undergoing TAVR, predictive factors of PPI after TAVR and outcomes over a large period of inclusion and follow-up.

## 2. Materials and Methods

### 2.1. Study Population and Setting

This retrospective, single-center, observational study was conducted at the Clairval Private Hospital (Marseille, France). All consecutive patients undergoing TAVR for severe, symptomatic aortic valve stenosis from 1 June 2012 to 31 June 2019 were recruited. Exclusion criteria included the presence of a pacemaker at admission and valve-in-valve procedures. Demographic and clinical data were acquired from the patient’s medical files, as well as electrocardiographic, echocardiographic, and cardiac computed tomography data before and after the TAVR procedure. The data set was abstracted in Excel (Excel 2016, Microsoft Corporation, Redmond, WD, USA) for analysis. Outcomes and survival follow-up data were collected at one month and each year. The Strengthening the Reporting of Observational Studies in Epidemiology (STROBE) reporting guideline was followed (https://www.strobe-statement.org (accessed on 4 January 2023)). This study is declared on the Health Data Hub registry, according to French legislation. The protocol was reviewed and approved by our Institutional Review Board (IRB number: 00010835). Informed consent was obtained from each patient, and data collection was performed anonymously.

### 2.2. Definitions and Outcomes

Transcatheter heart valve (THV) type was divided into the following two groups: balloon-expandable (Edwards Sapien™, Sapien XT™, and Sapien 3™ valves systems; Edwards Lifesciences, Irvine, CA, USA) and self-expandable valve [Medtronic CoreValve™, Evolut R™ systems (Medtronic, Minneapolis, MN, USA), and Accurate neo™ valve system (Boston Scientific, Marlborough, MA, USA)]. The primary outcome of this study was to assess the necessity of PPI within 30 days after TAVR, among demographic, comorbidity, electrocardiographic, clinical, anatomic, and procedural factors. Secondary outcomes included indications for PPI and survival. We also described the evolution of our practices over the study period. The oversizing degree was calculated using the following formula in patients who received self-expandable valves (nominal valve perimeter−measured perimeter)/nominal valve perimeter) × 100, and as follows for balloon-expandable valves (nominal valve area-measured area)/nominal valve area) × 100 [8]. The manufacturer provided the nominal valve perimeter and area. Chronic kidney disease was defined by a glomerular filtration rate < 60 mL/m^2^, and chronic respiratory disease by preoperative maximum respiratory volume per second (VEMS) < 50%. Finally, after an electrophysiological study, a threshold for the HV interval > 70 ms was used to indicate a PPI. Patients were described as having a third-degree AV block if this conductive disorder was sustained > 24 h after the procedure or if there was no underlying rhythm justifying urgent PPI after TAVR. No specific protocols were established to assess the need for pre-TAVR temporary pacemaker implantation or post-TAVR PPI, and a multidisciplinary evaluation was conducted for each patient.

### 2.3. Statistical Analysis

Categorial data are presented as numbers (%). Continuous variables are expressed as the means with standard deviations or medians with interquartile ranges (IQRs) with minimum and maximum values depending on the variable distribution. Patients were divided into the following two groups: with or without PPI at 30 days post-TAVR. In addition, we divided the study period into terciles (2012 to 2014, 2015 to 2017, and 2018 to 2019) to analyze variation over time. The factors associated with PPI were identified using multivariate forward stepwise logistic regression analysis, following previous univariate tests (Student’s *t*-test, the Mann–Whitney or Kruskal–Wallis was used to compare continuous variables, depending on their distribution, and the chi-square test or Fisher’s exact test was used to compare categorical variables). To analyze the prognostic effect on overall mortality at follow-up, univariate and multivariate stepwise logistic regression analyses were carried out. The variables tested were demographic characteristics (age, sex, BMI, NHYA classification), medical history (history of atrial fibrillation, chronic kidney disease, coronary artery disease, cerebrovascular disease, diabetes, dyslipidemia, hypertension, coronary angioplasty, coronary bypass surgery, myocardial infarction within 90 days, atrial fibrillation, chronic respiratory disease, obliterative arteriopathy in lower limbs), baseline conduction or rhythm disturbances (right bundle branch block [RBBB], left bundle branch block [LBBB]), incomplete LBBB (QRS duration between 110 and 119 ms), left anterior hemiblock (first-degree AV block, atrial fibrillation [AF] or flutter), and procedural characteristics (valve type, and oversize index). Variables were retained for the logistic regression if the *p*-value of the univariate test was <0.1. Further selection was based on clinical reasoning. The odds ratio was generated for all prediction values, and a significant level of *p* < 0.05 was used to indicate statistical significance. Kaplan–Meier analysis was performed for survival analysis to provide survival estimates, which were evaluated with a log-rank test. A 2-sided *p*-value of < 0.05 was considered statistically significant. All analyses were performed using the SAS release 9.6 (SAS Institute Inc., Cary, NC, USA).

## 3. Results

### 3.1. Study Population and Procedural Characteristics

**Figure 1:**
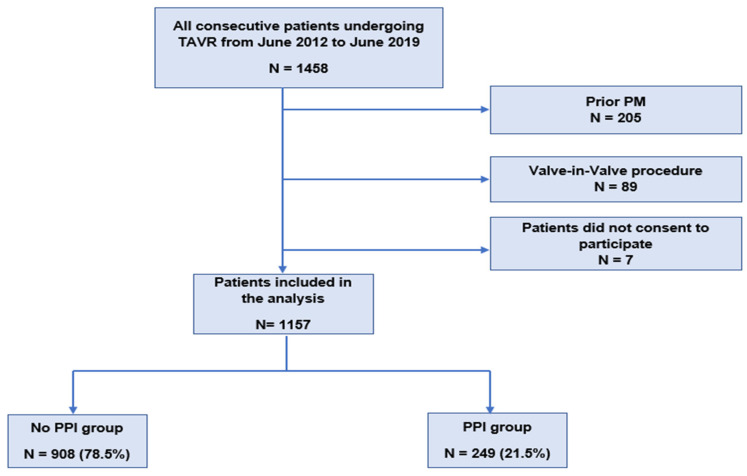
Flowchart of the study population. PM: pacemaker; PPI: permanent pacemaker implantation; and TAVR: transcatheter aortic valve replacement.

Among 1458 consecutive patients who underwent TAVR during the study period, 205 patients were excluded because of prior pacemakers, 89 because they had undergone a valve-in-valve procedure, and 7 patients did not consent to the inclusion. Ultimately, 1157 patients were included in the analysis. The median follow-up time was 44.9 months (95% CI: 43–47.2). The flowchart of the study population is described in Figure 1. The baseline and procedural characteristics of the population are summarized in Table 1. The mean age was 82.01 ± 6.9 years and 592 patients (52%) were women. The mean logistic EuroSCORE was 16.71 ± 11.06. The mean aortic gradient was 50.5 ± 14 mmHg. The transfemoral approach provided the most frequent access route for TAVR, performed in 74.6% of cases. The peri and post-procedural characteristics are described in Table 2. One hundred and thirty-one patients (11.3%) had self-expandable THV whereas 1025 (88.6%) had balloon-expandable THV. Post-procedural paravalvular aortic regurgitation was mild in 346 patients (30.6%), moderate in 48 patients (4.2%), and severe in 4 patients (0.4%). Twenty patients (1.7%) had a stroke post-procedure, 6 patients (0.6%) had a myocardial infarction, and pericardial tamponade occurred in 18 patients (1.6%). No patient had valve migration. The mean hospital stay after TAVR was 10 ± 6 days.

### 3.2. PPI and Predictive Factor

PPI was required in 249 patients (21.5%). The median time between TAVR and PPI was 4 days (IQR: 3–5). The main indications for PPI were: third-degree atrioventricular (AV) block (46.4%), followed by LBBB with pathological HV (24.6%), and LBBB without electrophysiological exploration (21.4%). The mean oversize index was estimated at 13.8 ± 12.2% in the PPI group and 12.6 ± 10.3% in the non-PPI group (*p* = 0.99). The variables significantly associated with PPI in univariate and multivariate analysis are presented in Table 3. Baseline RBBB (ORadj 2.49, 95% CI 1.44 to 4.30; *p* = 0.001), longer baseline QRS duration (ORadj 1.01, 95% CI 1.00 to1.02, *p* = 0.002), and self-expandable valves (ORadj 1.82, 95% CI, 1.09 to 3.03; *p* = 0.021) were found to be independent predictive factors of PPI.

### 3.3. Change in Practices over Time

An overview of the evolution of practices during the study period is provided in Table 4. The PPI incidence rate was 29.5% from 2012 to 2014 and gradually decreased over time as follows: 24.2% between 2015 and 2017 and 13.7% between 2018 and 2019 (*p* < 0.0001). The mean oversize index evolved significantly between 2012 and 2019. It was estimated to be 15.21 ± 16.7% from 2012 to 2014 and gradually decreased over time to 13.68 ± 8.3% (from 2015 to 2017) and 10.36 ± 9.8% (from 2018 to 2019), respectively (*p* < 0.0001). The transfemoral approach was the most frequent access route, whatever the period, and peaked in the 2018–2019 period (86.3%). Balloon-expandable valves implantation peaked implanted in the 2015–2017 period, whereas self-expandable valves implantation peaked over the 2012–2014 period (28.6%) (*p* < 0.001).

### 3.4. Survival Analysis

**Figure 2:**
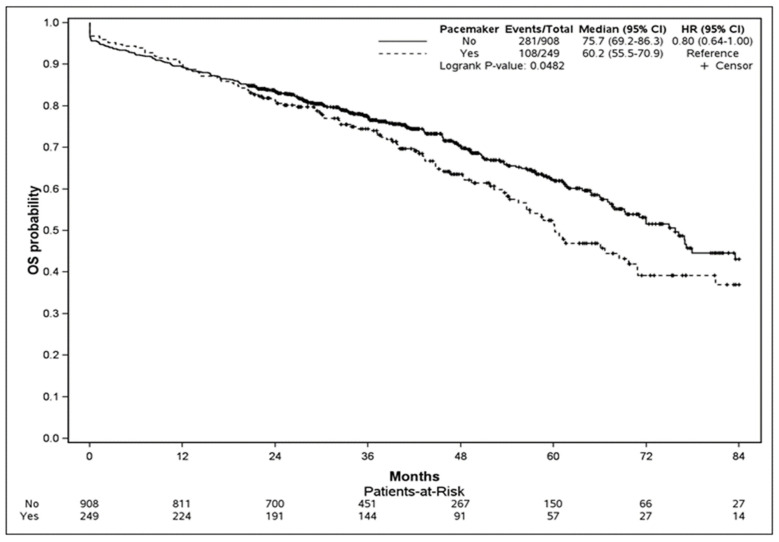
Kaplan-Meier curves comparing survival stratified by PPI and the non-PPI group. The test comparing the two groups was based on the log-rank test.

Overall, the all-cause estimated mortality rate was 58% (95% CI: 53–64) at 7 years. During follow-up, mortality was higher in patients with PPI (43.3%) compared to those without (30.9%) (log-rank test, *p* = 0.048). Kaplan–Meier survival analysis comparing mortality according to the presence of PPI is presented in Figure 2. The univariate and multivariate analyses of factors associated with mortality are described in Table 5. After adjustment, PPI (ORadj 2.49, 95% CI 1.4 to 4.3; *p* = 0.002), chronic kidney disease (ORadj 1.53, 95% CI 1.11 to 2.12; *p* = 0.01), chronic respiratory disease (ORadj 1.45, 95% CI 1.04 to 2.03; *p* = 0.03), obliterative arteriopathy in the lower limbs (ORadj 1.38, 95% CI 1.02 to 1.87; *p* = 0.04), and AF (ORadj 1.95, 95% CI 1.4 to 2.71; *p* < 0.0001) were the only remaining factors associated with death. Survival according to PPI indication was not different between third-degree AV block, LBBB with pathological HV, and LBBB (Supplementary Figure S1).

## 4. Discussion

The key findings of this study, which involved a large single-center cohort of patients undergoing TAVR with an extended follow-up of up to 7 years, can be summarized as follows: (i) the rate of PPI remained notably high at 21.5%, and its incidence decreased over time. (ii) Three independent predictive factors for PPI were identified as follows: a longer baseline QRS duration, the presence of RBBB at the baseline, and the use of self-expandable valves. (iii) Patients in the PPI group exhibited higher long-term mortality compared to their counterparts. (iv) PPI emerged as an independent predictive factor for long-term mortality, along with other factors, such as chronic kidney disease, chronic respiratory disease, obliterative arteriopathy, and AF.

In this study, the rate of PPI was elevated at 21.5%, with a higher prevalence observed in patients receiving self-expandable valves (35.6%). While this rate exceeded that of contemporary cohorts [9,10,11], the range across the literature was highly variable, ranging from 3.4% to 25.9%, according to the 2021 guidelines on cardiac pacing [12]. Several factors contributed to this variability. Firstly, the included patients might carry a higher risk of conduction disorders based on their profiles, as evidenced by PPI rates of 8.1% in the intermediate-risk cohort and 6.5% in the low-risk cohort of the PARTNER trials [13,14]. Secondly, this rate appeared to be dependent on the type of valve used, with self-expandable valves consistently associated with higher rates compared to balloon-expandable valves. Thirdly, variations in indications and protocols among different centers can significantly influence the PPI rate. For instance, in our cohort, less than half of the patients had a third-degree block warranting PPI, and the majority of implanted patients had LBBB. However, indications for pacemaker implantation in LBBB post-TAVR are a subject of debate, and current guidelines exhibit a liberal stance, suggesting electrophysiology studies or ambulatory ECG monitoring with the same level of evidence (Class IIa). These findings hold clinical significance for our daily practice and underscore the imperative need for standardized protocols and further evidence to determine the optimal approach for this substantial patient population.

We found a robust association between PPI and the presence of RBBB (OR 2.51, 95% CI 1.45–4.36). The link between conduction disorders and the need for PPI following TAVR is a well-established association, with pre-existing RBBB consistently identified as the most significant and powerful contributing factor [15,16]. The proximity of the conduction pathways, especially the left bundle to the aortic valve, makes them susceptible to injury during aortic prosthesis deployment through various mechanisms—with hemorrhagic, compressive, and ischemic effects on the conductive tissue. These mechanisms result in high-grade conduction disorders and complete atrioventricular block. Additionally, other conduction disorders and the use of self-expandable valves are also found to be associated with PPI, aligning with the existing literature available [17,18]. One aspect not explored in our study, yet notably associated with PPI, is the depth of valve implantation. This parameter is gaining recognition, and operators are employing various techniques, such as deploying the valve in the cusp overlap view, to position the valve at the highest reachable level [19,20]. However, concerns about future coronary re-access in cases of the high implantation of THV are now emerging [19]. Thus, few modifiable risks are currently identified, with the majority being established as non-modifiable risk factors (preoperative conductive disorders). While these data allow for the prediction of risk, they may not necessarily lead to its prevention. The solution might lie not only in improving practices but also in enhancing valves to minimize trauma post-deployment. Nevertheless, achieving very low rates of pacemaker implantation in this type of procedure remains challenging. In our study, although we observed a higher proportion of women requiring pacemaker implantation after TAVI, this factor was not predictive in multivariate analysis. This observation contrasts with the literature, where women are often described as less frequently requiring pacemaker implantation [21].

To the best of our knowledge, this study represents one of the largest single-center TAVR registries in France, offering an extensive long-term follow-up. This prolonged observation period enabled the identification of robust predictors of mortality. Among these factors, PPI emerged as the most significant, exhibiting a two-fold increase in mortality. This phenomenon may be attributed to various factors. Firstly, patients with indications for PPI may be more likely to have comorbidities, and PPI could serve as a marker of frailty. Secondly, right ventricular stimulation has been linked to heart failure hospitalization and mortality, even in individuals without pre-existing health issues [20]. This association is tied to electromechanical dyssynchrony. These findings emphasize the non-trivial impact of PPI on patients and underscore the caution required in its indication. Furthermore, when the indication is confirmed, physiological pacing options, such as bundle pacing or left bundle branch pacing, should be considered. Additionally, given the low rates of long-term pacing dependency, especially in patients with LBBB, algorithms promoting spontaneous atrioventricular conduction should be employed. Moreover, the increasing utilization of leadless pacemakers as a safe alternative for high-risk patients has generated significant interest. Some studies have highlighted safety and efficacity, supporting their utilization [22]. However, recent findings underscore potential concerns, with evidence suggesting higher inhospitality rates compared to traditional transvenous devices [23]. Given these conflicting findings, larger randomized trials are essential to elucidate the true benefits and risks associated with the use of leadless pacemakers in this setting.

### Study Limitations

Our analysis has several limitations. The primary constraint arises from the retrospective and single-center nature of this study, introducing biases inherent to the methodology and associated with data collection in a registry. The presence of unmeasured confounders cannot be ruled out. Furthermore, certain variables known to be predictive factors of PPI, such as the implantation depth and modality of valve deployment, were not recorded. Only life status was reported during follow-up, and information on re-hospitalization for heart failure or the cause of death was not available. Lastly, we did not investigate long-term PPI dependency and had no access to specific data concerning the programming and pacing parameters of the pacing devices. It is important to note that the absence of data on the burden of pacing limits our ability to ascertain the true population of patients who genuinely require a pacemaker. The inability to identify cases where the device was truly necessary underscores the importance of a more precise evaluation of indications. It is plausible that a subset of patients may have been exposed to increased mortality without a confirmed need for a pacemaker, highlighting the necessity for enhanced caution in defining indications.

## 5. Conclusions

This monocentric study highlights elevated rates of PPI post-TAVR, reaching 21.5%, associated with increased mortality in affected patients. These findings underscore the urgent need to reassess our practices, refine indications, and optimize the future outlook for implanted patients, particularly in reducing high PPI rates and improving long-term outcomes.

## Figures and Tables

**Table 1:** Baseline and preprocedural characteristics of the study population (n = 1157): patients with or without 30-day permanent pacemaker implantation (PPI) after TAVR.

	Whole Cohort (n = 1157)	No PPI Group (n= 908)	PPI Group (n = 249)	*p* Value
Age, y, mean ± SD	82.02 ± 6.98	81.78 ± 6.97	82.89 ± 6.96	0.015
Female, n (%)	592 (52.1)	479 (53.6)	113 (46.3)	0.042
BMI, kg/m^2^, mean ± SD	26.37 ± 4.58	26.33 ± 4.58	26.51 ± 4.60	0.825
EuroSCORE, mean ± SD	16.71 ± 11.06	16.51 ± 10.94	17.43 ± 11.50	0.249
Cardiovascular risk factors				
Hypertension, n (%)	736 (63.6)	575 (63.3)	161 (64.7)	0.699
Dyslipidaemia, n (%)	404 (34.9)	320 (35.2)	84 (33.7)	0.659
Non-insulin-dependent diabetes mellitus, n (%)	262 (22.6)	202 (22.2)	60 (24.1)	0.537
Insulin-dependent diabetes mellitus, n (%)	75 (6.5)	60 (6.6)	15 (6)	0.740
Current smoker, n (%)	40 (3.5)	34 (3.7)	6 (2.4)	0.307
Coexisting illnesses				
Chronic kidney disease (GFR < 60 mL/m^2^), n (%)	334 (29)	251 (27.8)	83 (33.3)	0.090
Dialysis, n (%)	33(2.9)	31 (3.4)	2 (0.8)	0.0295
Coronary artery disease, n (%)	582 (51.1)	445 (49.8)	137 (55.7)	0.104
Chronic respiratory disease (VEMS < 50%), n (%)	296 (26.1)	231 (26)	65 (26.4)	0.897
Percutaneous coronary intervention, n (%)	454 (39.2)	345 (38)	109 (43.8)	0.098
Cerebrovascular disease, n (%)	95 (8.2)	73 (8.1)	22 (8.8)	0.696
Obliterative arteriopathy in lower limbs, n (%)	437 (38.5)	345 (38.8)	92 (37.6)	0.720
Atrial fibrillation, n (%)	291 (25.3)	216 (23.9)	75 (30.4)	0.038
Mitral regurgitation (grade 3+), n (%)	28 (2.4)	25 (2.8)	3 (1.2)	0.241
Coronary artery bypass surgery, n (%)	72 (6.2)	53 (5.8)	19 (7.6)	0.301
Previous aortic valve valvuloplasty, n (%)	183 (15.9)	141 (15.6)	42 (16.9)	0.624
LVEF, %, mean ± SD	57 ± 11.8	57.3 ± 11.8	55.9 ± 11.8	0.052
Cardiac symptoms, N (%)				
NYHA functional scale				
I–II	296 (26.1)	243 (27.3)	53 (21.5)	0.65
III–IV	839 (74)	646 (72.6)	193 (78.5)	0.65
Syncope	45 (3.9)	35 (3.9)	10 (4)	0.907
Hearth failure	6 (0.5)	4 (0.4)	2 (0.8)	0.615
Angina	8 (0.7)	7 (0.8)	1 (0.4)	1
Pre-procedure electrocardiographic variables				
QRS duration, ms, mean ± SD	99.9 ± 23.8	97.4± 22.6	108.9 ± 25.9	<0.0001
PR interval ms, mean ± SD	176.8 ± 44.1	175.4 ± 42.5	181.8 ± 49.6	0.069
Sinus rhythm, n (%)	946 (81.8)	747 (82.3)	199 (79.9)	0.395
LVH, n (%)	8 (0.7)	7 (0.8)	1 (0.4)	1
First-degree AVB, n (%)	172 (14.9)	128 (14.1)	44 (17.7)	0.160
LBBB, n (%)	107 (9.2)	79 (8.7)	28 (11.2)	0.22
RBBB, n (%)	116 (10)	61 (6.7)	55 (22.1)	<0.001
Left anterior hemiblock, n (%)	57 (4.9)	38 (4.2)	19 (7.6)	0.026
Incomplete left bundle branch block, n (%)	11 (1)	6 (0.7)	5 (2)	0.052
Atrial fibrillation, n (%)	159 (13.7)	113 (12.4)	46 (18.5)	0.014
Atrial flutter, n (%)	157 (13.6)	111 (12.2)	46 (18.5)	0.0107
Bradycardia, n (%)	2 (0.2)	1 (0.1)	1 (0.4)	0.384
Pre-procedure echocardiographic variables				
Aortic-valve gradient, mmhg, mean ± SD	50.5 ± 14.3	50.8 ± 14.3	49.6 ± 14.6	0.074
Aortic-valve area, cm^2^, mean ± SD	0.957 ± 3.967	1.017 ± 4.454	0.729 ± 0.473	0.594
PAP, mmhg, mean ± SD	24.9 ± 24.2	29.8 ± 35.9	26.0 ± 27.3	0.078
Baseline treatment, N (%)				
Amiodarone	174 (26.3)	117 (22.9)	57 (38)	<0.001
β-blocker	424 (46.5)	331 (45.6)	93 (50)	0.282
Digoxin	15 (3)	12 (2.9)	3 (3.1)	1
Flecainide	18 (3.6)	16 (3.9)	2 (2.1)	0.547
Calcium channel antagonists	49 (9.1)	39 (9)	10 (9.7)	0.818

AVB: atrioventricular block; GFR: glomerular filtration rate; LBBB: left bundle branch block; LVEF: left ventricular ejection fraction; LVH: left ventricular hypertrophy; PAP: pulmonary artery pressure; PPI: permanent pacemaker implantation; RBBB: right bundle branch block; and VEMS: maximum expiratory volume per second.

**Table 2:** Periprocedural and postprocedural characteristics.

	Whole Cohort (n = 1157)	No PPI Group (n= 908)	PPI Group (n = 249)	*p* Value
Periprocedural characteristics, N (%)				
Access route				
Transfemoral access	862 (74.6)	669 (73.7)	193 (77.8)	0.107
Trans aortic access	154 (13.3)	124 (13.7)	30 (12.1)	0.107
Trans carotid access	87 (7.5)	76 (8.1)	11 (4.4)	0.107
Transapical access	52 (4.5)	38 (4.2)	14 (5.6)	0.107
Prosthetic valve type				
Balloon-expandable valve	1025 (98.6)	820 (90.4)	205 (82.3)	<0.001
Self-expandable valve	131 (11.3)	87 (9.5)	44 (17.6)	<0.001
Nominal area oversizing, %, mean ± SD	12.89 ± 10.77	12.64 ± 10.32	13.77 ± 12.18	0.993
Electrocardiographic variables post-TAVR				
QRS duration, ms, mean ± SD	117.3 ± 52.9	109.4 ± 54.9	150.4 ± 23.6	<0.0001
PR interval, ms, mean ± SD	186.4 ± 62.6	182.9 ± 56.8	202.5 ± 82.4	<0.0001
Complications N (%)				
Stroke	20 (1.7)	16 (1.8)	4 (1.6)	1
Myocardial infarction	6 (0.6)	5 (0.6)	1 (0.4)	1
Valve migration	4 (100)	4	0	-
Periprosthetic regurgitation				
Grade 0 (no regurgitation)	733 (64.8)	567 (64.1)	166 (67.5)	0.627
Grade 1 (Mild)	346 (30.6)	278 (31.4)	68 (27.7)	0.627
Grade 2 (Moderate)	48 (4.2)	37 (4.2)	11 (4.5)	0.627
Grade 3 and 4 (Severe)	4 (0.4)	3 (0.3)	1 (0.4)	0.627
Severe vascular access complications according to VARC 3 criteria	55 (5.2)	42 (5)	13 (5.8)	0.634
In-hospital death, n (%)	42 (3.6)	37 (4.1)	5 (2)	0.122
Length of hospital stay, days, mean ± SD	10.1 ± 6.3	9.6 ± 6.2	11.7 ± 6.4	<0.0001
Estimated one-year mortality (95%, IC), %	11 (9–12)	11 (9–13)	10 (7–15)	0.749 *
Estimated 7-year mortality (95%, IC), %	58 (53–64)	57 (50–65)	63 (54–73)	0.048 *

* by log-rank test.

**Table 3:** Independent factors for 30-day permanent pacemaker implantation after transcatheter aortic valve replacement.

	Univariable Analysis		Multivariable Analysis	
Total Population	OR (95% CI)	*p* Value	OR (95% CI)	*p* Value
Creatinine serum level	1.001 (0.99–1.02)	0.894		
History of AF	1.38 (1.02–1.90)	0.039		
Baseline electrocardiographic variables				
AF	1.62 (1.11–2.37)	0.011		
Flutter	2 (1.42–2.81)			
RBBB	3.94 (2.64–5.85)	<0.0001	2.49 (1.4–4.30)	0.001
Left anterior hemiblock	1.89 (1.07–3.34)	0.028		
Longer baseline QRS duration	1.019 (1.013–1.025)	<0.0001	1.01 (1.004–1.02)	0.0028
Echocardiographic and CT scan variables				
Aortic annulus diameter in Echocardiography	1.104 (1.026–1.187)	0.008		
Aortic annulus diameter in CT scan	1.125 (1.054–1.202)	0.0004		
Baseline treatment				
Preoperative amiodarone	2.07 (1.4–3.05)	0.0002		
Valve type				
Self-expandable valves	2.02 (1.36–3.00)	0.0004	1.82 (1.09–3.04)	0.021
Post-TAVR electrocardiographic variables				
QRS duration (ms)	1.040 (1.03–1.05)	<0.0001		
PR interval (ms)	1.005 (1.00–1.01)	0.0003		

**Table 4:** Evolution of practices over time.

	2012–2014 (n = 220)	2015–2017 (n = 528)	2018–2019 (n = 409)	*p* Value
Access route				
Transfemoral, n (%)	127 (58)	407 (77.5)	327 (86.3)	-
Trans aortic, n (%)	63 (28.8)	80 (15.2)	11 (2.9)	-
Trans carotid, n (%)	0 (0)	4 (0.8)	19 (5)	-
Transapical, n (%)	25 (11.4)	21 (4)	5 (1.3)	-
Type of valve				
Balloon-expandable valve, n (%)	157 (71.4)	503 (95.3%)	365 (89.5)	<0.001
Self-expandable valve, n (%)	63 (28.6)	25 (4.7)	43 (10.6)	<0.001
Nominal area oversizing, %, mean ± SD	15.2 ± 16.7	13.7 ± 8.3	12.9 ± 10.7	<0.0001
PPI rate, n (%)	65 (29.5)	128 (24.2)	56 (13.7)	<0.001
PPI indication				
third-degree atrioventricular block, n (%)	16 (32)	63 (51.6)	25 (48.1)	<0.001
LBBB with pathological electrophysiologic exploration, n (%)	9 (18)	24 (19.7)	22 (42.3)	<0.001
LBBB without electrophysiologic exploration, n (%)	18 (36)	29 (23.8)	1 (1.9)	<0.001
Second-degree atrioventricular block, n (%)	1 (2)	3 (2.5)	2 (3.8)	<0.001
Others, n (%)	4 (8)	3 (2.5)	1 (1.9)	<0.001
Complications N (%)				
Stroke	3 (1.4)	11 (2.1)	6 (1.5)	0.80
Myocardial infarction	2 (0.9)	3 (0.6)	1 (0.3)	0.43
Valve migration	0	3 (0.75)	1 (0.25)	-
Periprosthetic regurgitation				
Grade 0 (no regurgitation)	95 (44%)	387 (73.6%)	251 (64.5%)	<0.001
Grade 1 (mild)	100 (46.3%)	130 (24.7%)	116 (29.8%)	<0.001
Grade 2 (moderate)	20 (9.3%)	8 (1.5%)	20 (5.1%)	<0.001
Grade 3 and 4 (Severe)	1 (0.5%)	1 (0.2%)	2 (0.5%)	<0.001
In-hospital death, n (%)	14 (6.4)	22 (4.2)	6 (1.5)	0.005
Length of hospital stay, days, mean ± SD	13.4 ± 9.2	9.5 ± 5	9 ± 5.1	<0.0001

LBBB: left bundle branch block.

**Table 5:** Independent risk factors of overall mortality at logistic regression analysis.

	Univariable Analysis		Multivariable Analysis	
Total Population	OR (95% CI)	*p* Value	OR (95% CI)	*p* Value
Age	1.021 (1.003–1.04)	0.023		
Female	0.896 (0.7–1.15)	0.382		
Coexisting illness				
Chronic kidney disease	1.74 (1.33–2.26)	<0.0001	1.53 (1.11–2.12)	0.01
Chronic respiratory disease	1.58 (1.2–2.08)	0.001	1.45 (1.04–2.03)	0.03
Dialysis	2.75 (1.36- 5.56)	0.005		
Mitral regurgitation	2.32 (1.09–4.93)	0.028		
Obliterative arteriopathy in lower limbs	1.39 (1.07–1.78)	0.011	1.38 (1.02–1.87)	0.04
Cardiac symptoms				
NYHA functional disease				
I-II	1.397 (0.626–3.116)	0.414		
Syncope	2.067 (0.990–4.356)	0.053		
ECG variables at baseline				
Sinus Rhythm	1.86 (1.37–2.52)	<0.0001		
Narrow QRS complex (<120 ms)	1.61 (1.23–2.09)	0.0005		
AF	2 (1.43–2.78)	<0.0001	1.95 (1.4–2.71)	<0.001
Access route				
Trans aortic	3.768 (2.642–5.375)	<0.0001		
Trans carotid	0.520 (0.175–1.545)	0.239		
Transapical	1.875 (1.057–3.327)	0.032		
Valve type				
Balloon-expandable valve	0.718 (0.495–1.042)	0.0809		
Nominal area oversizing	1.02 (0.97–1.07)	0.527		
Need for PPI	1.71 (1.28–2.28)	0.0003	2.49 (1.4–4.3)	0.002

AF: atrial fibrillation; CI = confidence interval; OR = odds ratio; and PPI: permanent pacemaker implantation.

## Data Availability

Data are contained within the article.

## Supplementary Materials

The following supporting information can be downloaded at: https://www.mdpi.com/article/10.3390/jcm13113050/s1, Figure S1: Kaplan-Meier curves comparing survival stratified by third-degree atrioventricular block and no third-degree atrioventricular block in the PPI group. The test comparing the two groups was based on the log-rank test.
