# Supplementary material for: Prognosis and Predictor Factors of Permanent Pacemaker Implantation after Transcatheter Aortic Valve Replacement: A Retrospective Analysis of the Post-Transcatheter Aortic Replacement Clairval Hospital Registry
Source: J Clin Med. 2024 May 22;13(11):3050. doi: 10.3390/jcm13113050 (PMC11173049; doi:10.3390/jcm13113050)
Supplement: Supplementary file 1 [file jcm-13-03050-s001.zip › jcm-3001214-supplementary.pdf]

**Supplemental Figure S1**

**Kaplan-Meier curves comparing survival stratified by AV3B and no AV3B group in the PPI group. Test comparing the two groups was based on the log-rank test.**

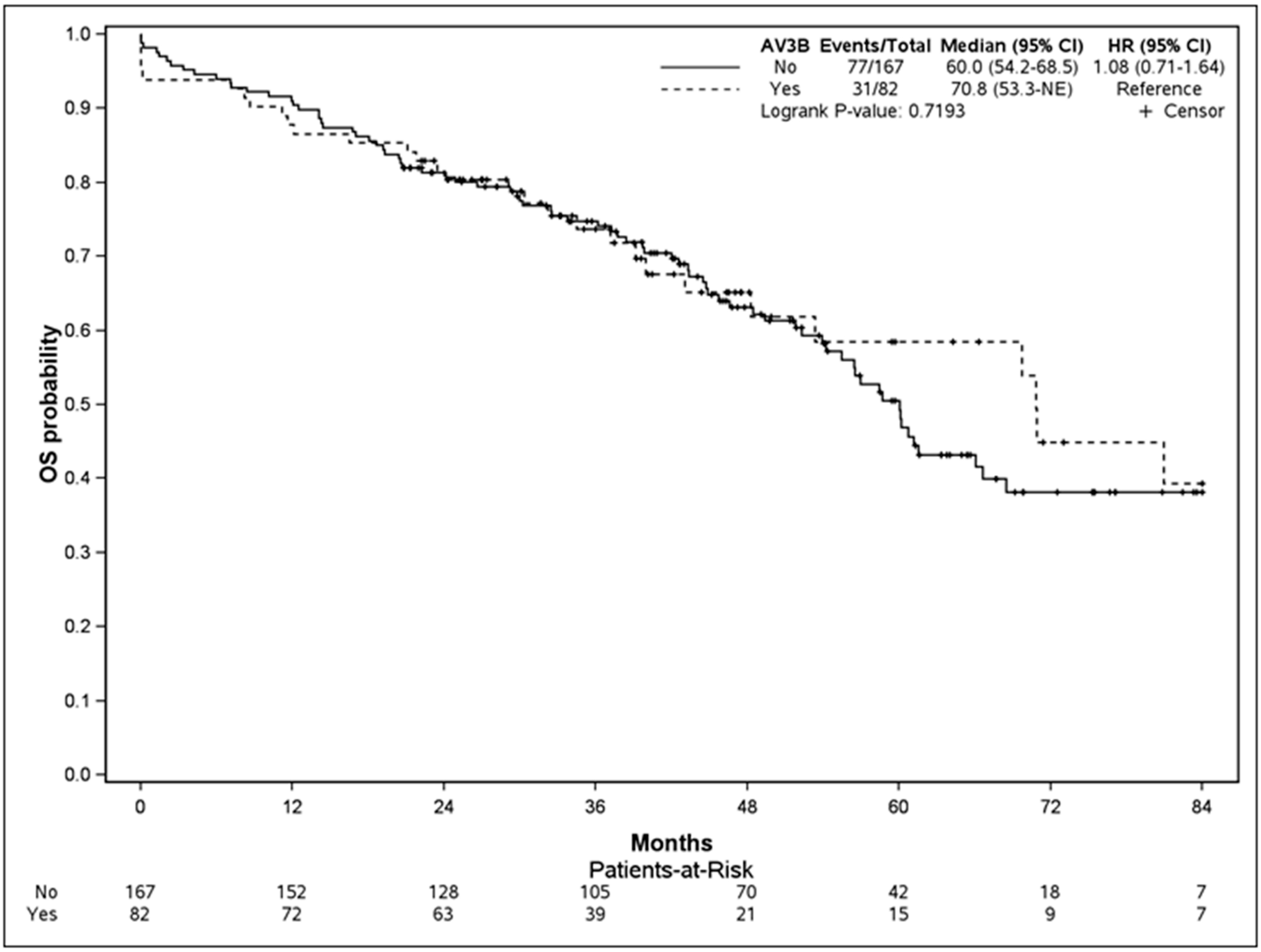


AV3B: third degree atrioventricular block; LBBB: left bundle branch block.

**Kaplan-Meier curves comparing survival stratified by LBBB and no LBBB group in PPI group. Test comparing the two groups was based on the log-rank test.**

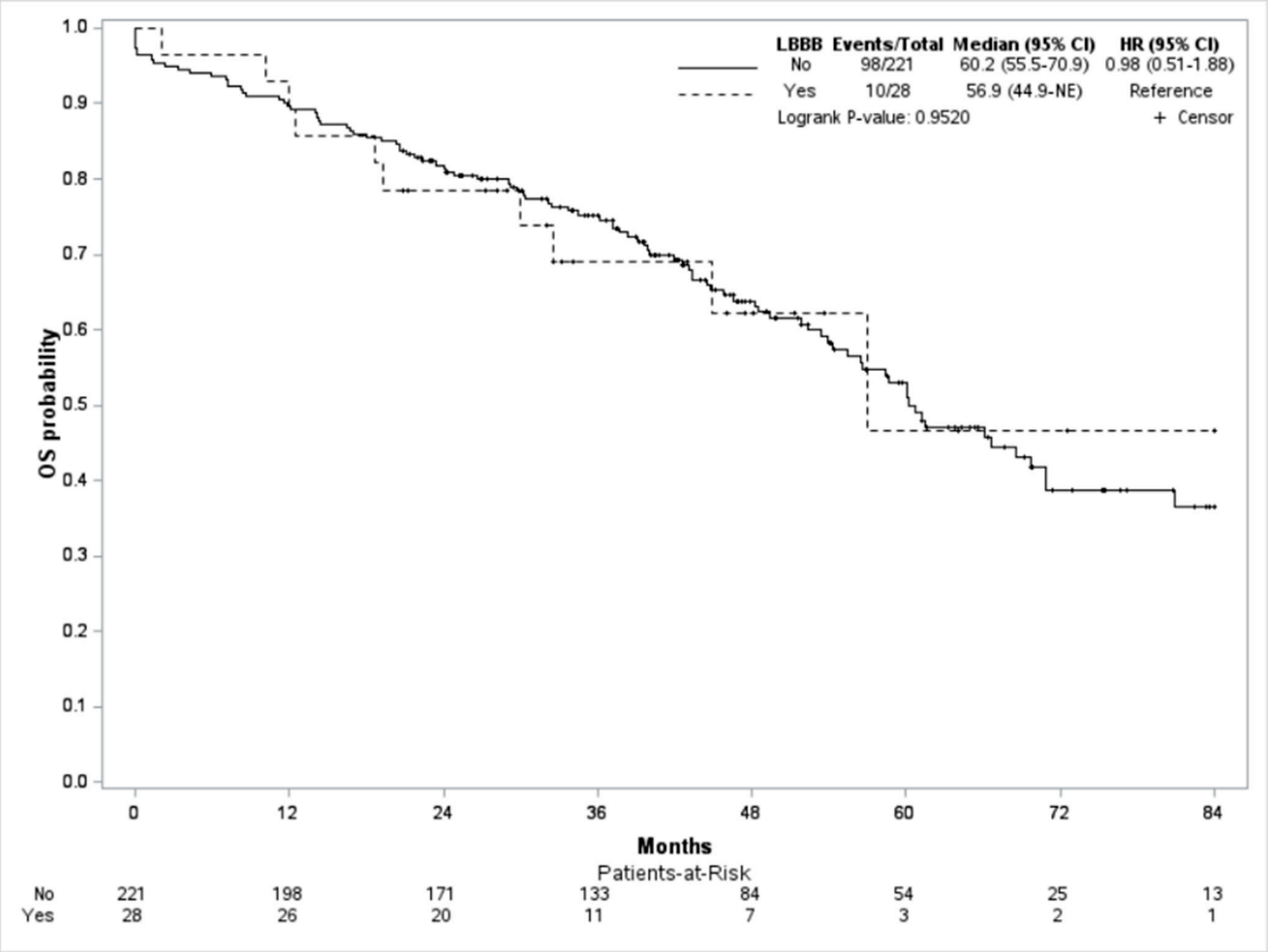


LBBB: left bundle branch block.

**Kaplan-Meier curves comparing survival stratified by LBBB with pathological HV and no LBBB with pathological HV group in PPI group. Test comparing the two groups was based on the log-rank test.**

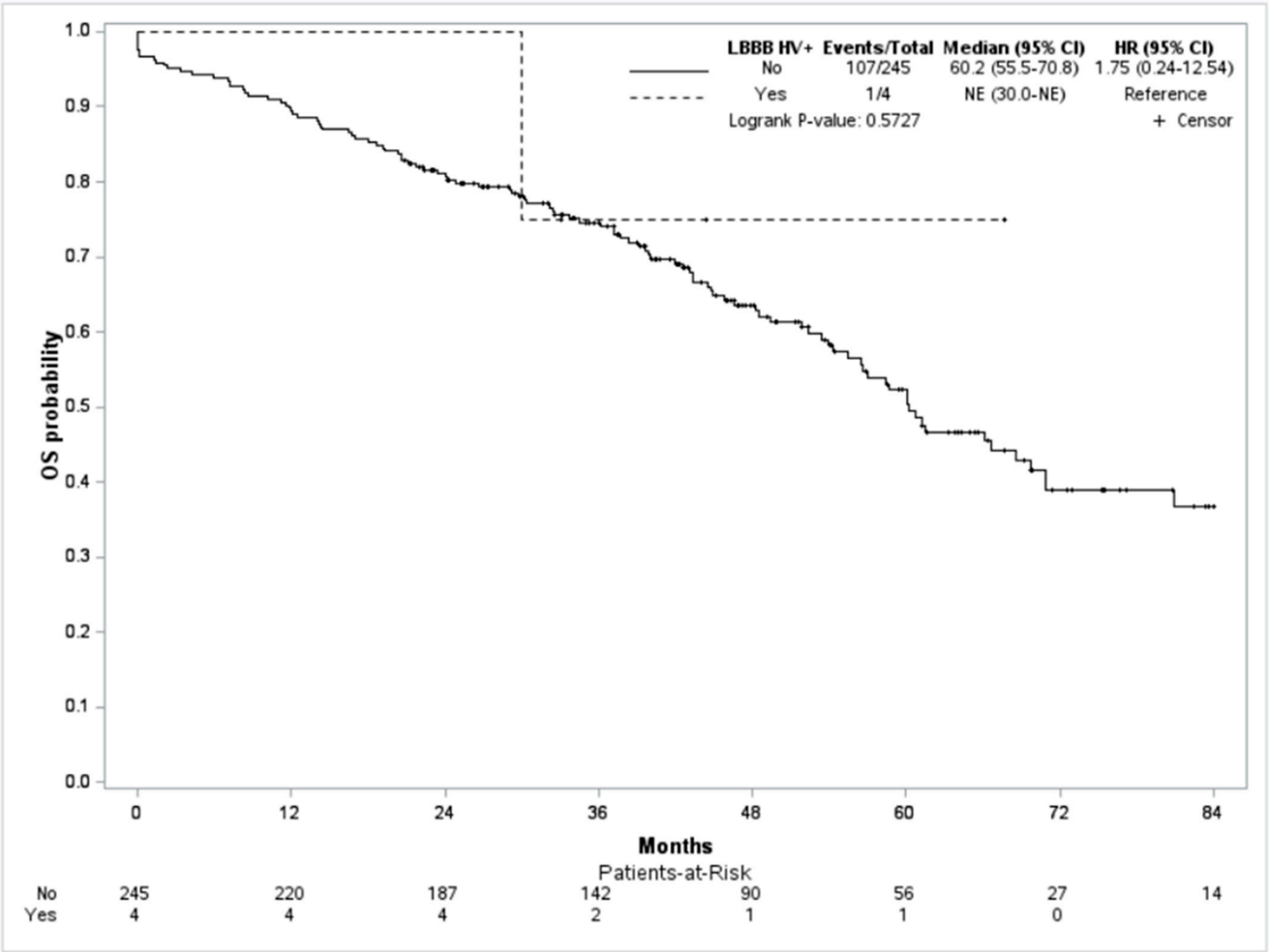


LBBB HV +: left bundle branch block with pathological HV-interval.
